# Interferon-alpha responsible EPN3 regulates hepatitis B virus replication

**DOI:** 10.3389/fmed.2022.944489

**Published:** 2022-07-22

**Authors:** Xueqian Li, Zhe Wang, Weiping Zhou, Xuanhe Fu, Yunpeng Zhang, Ye Sun, Biao Yang, Yuxin Bai, Chunwei Dai, Xiaolun Xu, Fan Cui, Ying Zhao, Yuping Zhang, Bengang Wang, Yingfang Li, Masamichi Muramatsu, Kousho Wakae, Guangyan Liu

**Affiliations:** ^1^Department of Pathogen Biology, Shenyang Medical College, Shenyang, China; ^2^Department of Medical Oncology, Affiliated Zhongshan Hospital of Dalian University, Dalian, China; ^3^The Key Laboratory of Biomarker High Throughput Screening and Target Translation of Breast and Gastrointestinal Tumor, Dalian University, Dalian, China; ^4^Department of Immunology, Shenyang Medical College, Shenyang, China; ^5^Department of Pathophysiology, Shenyang Medical College, Shenyang, China; ^6^College of Basic Medical Sciences, Shenyang Medical College, Shenyang, China; ^7^Department of Hepatobiliary Surgery, Institute of General Surgery, The First Hospital of China Medical University, Shenyang, China; ^8^Department of Virology II, National Institute of Infectious Disease, Tokyo, Japan; ^9^Foundation for Biomedical Research and Innovation, Kobe, Japan

**Keywords:** EPN3, HBV, IFN-α, p53, AID

## Abstract

Hepatitis B virus (HBV) infection remains a major health problem worldwide, and the current antiviral therapy, including nucleoside analogs, cannot achieve life-long cure, and clarification of antiviral host immunity is necessary for eradication. Here, we found that a clathrin-binding membrane protein epsin3 (EPN3) negatively regulates the expression of HBV RNA. EPN3 expression was induced by transfection of an HBV replicon plasmid, and reduced HBV-RNA level in hepatic cell lines and murine livers hydrodynamically injected with the HBV replicon plasmid. Viral RNA reduction by EPN3 was dependent on transcription, and independent from epsilon structure of viral RNA. Viral RNA reduction by overexpression of p53 or IFN-α treatment, was attenuated by knockdown of EPN3, suggesting its role downstream of IFN-α and p53. Taken together, this study demonstrates the anti-HBV role of EPN3. The mechanism how it decreases HBV transcription is discussed.

## Introduction

Hepatitis B is an infectious disease that remains as a major health problem worldwide (1, 2). Persistent infection with hepatitis B virus (HBV), an enveloped DNA virus, increases the risk of fibrosis, cirrhosis, and hepatocellular carcinoma (3–5). HBV RNAs, including pre-genomic (pg) RNA are transcribed from covalently closed circular DNA (cccDNA) in the nucleus. Two epsilon RNA structures are present in the 5′- and 3′- end of pgRNA, and the 5′-epsilon structure is especially important as a packaging signal (6). Epsilon and Pol protein are essential for pgRNA encapsidation, then pgRNA is reverse-transcribed to relaxed circular (RC)-DNA in the nucleocapsids. Nucleoside/nucleotide analogs (NA) such as lamivudine, adefovir, entecavir, telbivudine, and tenofovir are primarily used for HBV treatment (7, 8). Although NA has a healing effect on HBV patients, HBV DNA levels rebound upon discontinuation, forcing patients to life-long administration. This justifies alternative use of interferons, with some advantages such as long treatment duration, absence of drug resistance, delayed response, and higher hepatitis B surface antigen loss (9). Nonetheless, its use is limited due to its side effects deriving from its broad physiological effect. Thus, discriminating the antiviral activity of IFN-α from its side effects is necessary, to discover effective antiviral therapies with less side effects.

Epsins are evolutionarily conserved membrane protein family, which plays an important role in endocytosis and signal transduction of podocytes (10). The epsin family comprises of three members, Epsin1, Epsin2, and Epsin3 (EPN3), which share the N-terminal ENTH domain, 2 ubiquitin interacting motifs, clathrin binding motif, DPW and NPF motifs (11). EPN3 is reportedly involved in clathrin-mediated cell surface receptor internalization, and is demonstrated to mediate p53 signaling, inducing apoptosis in HCT116 cells (11), and senescence in U2OS cells (12). Moreover, Epsins are proposed to play tumorigenic roles such as angiogenesis, tumor cell migration, invasion, and epithelial-mesenchymal transformation (EMT) (13, 14). Nonetheless, its role in viral infection has not been investigated. Here, we investigated the role of EPN3 in HBV replication.

## Materials and methods

### Cell culture, plasmids, and transfection

Huh7 and HepG2 cells were cultured in Dulbecco's modified Eagle medium (DMEM; Biological Industries) supplemented with 10% fetal bovine serum (FBS; gibco) and 1% penicillin/streptomycin at 37°C in 5% CO_2_ atmosphere. The total amount of plasmids per sample was unified by supplementing empty plasmids. For transfection of plasmids and siRNAs, lipofectamine 2000 (Invitrogen) was utilized, according to the manufacturer's instruction. Interferon-alpha (IFN-α) and actinomycin D (ActD) were obtained from MedChemExpress and Sigma-Aldrich, respectively. siRNAs were purchased from RiboBio Company. The expression vectors used in this study are listed in Supplementary Table 1.

### RNA-seq

8 x 10^5^ of Huh7 cells were seeded on a 10 cm dish one day before transfection, and the next day, the HBV replicon plasmid (pPB) (15) or an empty vector (pcDNA3.1) was transfected in duplicate. The total RNA was isolated using Trizol Reagent (Invitrogen Life Technologies), and the sequencing libraries were generated utilizing TruSeq RNA Sample Preparation Kit (Illumina, San Diego, CA, USA), according to the manufacturer's protocols. Briefly, 3 μg of total RNA was subjected to poly-T oligo-attached magnetic beads to purify mRNA, enzymatically fragmented and reverse-transcribed. After second strand cDNA synthesis, blunting the overhangs, and adenylation of 3'-ends, Illumina PE adapter oligonucleotides were ligated and the fragments around 450 bp were selected using AMPure XP system (Beckman Coulter, Beverly, CA, USA). The selected fragments were further enriched by Illumina PCR Primer Cocktail, and validated by the Agilent high sensitivity DNA assay on a Bioanalyzer 2100 system (Agilent). The library was run on a Hiseq 2500 platform (Illumina) at 150 x 2 PE, by Shanghai Personal Biotechnology Cp. Ltd., and FASTQ files containing 97,297,560~136,396,736 reads per sample were obtained.

### RNA-seq data analyses

The FASTQ data was first subjected to Cutadapt (16) to obtain reads with high quality, and mapped to hg38 (ENSEMBLE) by HISAT2 (17). The mapped reads for each sample ranged from 86,503,474 to 110,678,626, accounting for 96.54%-97.21% of the initially obtained reads. The FPKM values were obtained by HTSeq (18), and genes affected by more than 2-fold with *p*-values <0.05 were extracted with DESeq (19). For the heatmap, genes were clustered based on the Euclidean distance and Complete Linkage method (20, 21).

### Luciferase assay

Luciferase analysis was performed as previously described in Que et al. (22). Briefly, the nano-luciferase (NL) activities of HBV-NL transfectants were determined using Nano-Glo Luciferase Kit (Promega) and PowerScan HT (BioTek). The NL gene was inserted into the core region of the HBV genome in the HBV-NL chimeric construct, and the NL activity represents pgRNA expression in the transfected hepatocyte cell line (22, 23). Samples were duplicated or triplicated within each biological replicate.

### Quantitative PCR

Cytoplasmic HBV DNA was purified as described in Gunther et al. (24) and Kitamura et al. (25), with minor modifications. Briefly, the cells were lysed with PBS containing 0.5% NP-40 and protease inhibitor cocktail (1x cOmplete, Roche), and the cytoplasmic supernatants were treated with DNase I, to remove the naked DNA. The capsids were lysed with 1% SDS, 10 mM EDTA, and 500 ug/mL proteinase K (Sigma-Aldrich), and the encapsidated DNA was extracted by phenol–chloroform extraction, followed by isopropanol precipitation. Secreted HBV DNA was also precipitated by PEG8000, followed by DNase I digestion and lysis in the buffer containing SDS, Tris-HCl (pH 8.0), EDTA, and proteinase K, for 4 h at 55°C.

Total RNA was extracted by TRIsure and treated with DNase I (Takara) to eliminate the transfected plasmids and genomic DNA. The DNase-treated RNA was reverse-transcribed with Reverse Transcription Kit (Vazyme) following the manufacturer's instruction. Quantitative PCR (qPCR) analysis was performed with ChamQ Universal SYBR qPCR Master Mix (Vazyme) on Real-Time PCR systems (ABI 7500). The HBV DNA copy numbers were absolutely quantified using the standard curve based on the pPB plasmid. The results were normalized by internal control β-actin, and the samples were triplicated within each biological replicate. The primer sequences are listed in Supplementary Table 2.

### Western blot

Western blot was performed as described previously (25, 26). Signals were visualized and captured by Odyssey or DNR Bio-imaging instrument. The antibodies used in this study are mouse anti-GAPDH (SolelyBio, TA08), mouse anti-ACTIN (Fude Biological, FD0060), rabbit anti-FLAG (Mannan Binding Lectin, PM020), rabbit anti-GFP (Cell Signaling Technology, 2956S), rabbit anti-EPN3 (CUSABIO, CSB-PA030170), rabbit anti-p53 (Immunoway, YT3528), anti-rabbit IgG-HRP (EnoGene, E1WP318/abcam, ab216777), and anti-mouse IgG-HRP (EnoGene, E1WP319/Thermo, A11375).

### Enzyme-linked immunosorbent assay (ELISA)

The supernatants were collected 72 h after transfection, and Hepatitis B surface antigen (HBsAg) level was determined as described in Jiang et al. (27), utilizing Human HBsAg ELISA KIT) (Lvye biotechnology) and TECAN infinite M200PRO, following the manufacturer's instruction.

### *In vivo* hydrodynamic-based plasmid delivery system

We followed the method described in Liu et al. (28). In brief, female C57BL/6 mice (6–10 weeks old, Changsheng biotechnology) were injected with pPB and the EPN3 expression vector, reconstituted in PBS, equivalent volume of 10% of the body weight, through the tail vein. The injection procedure was completed within 8 s, at the constant rate. All animal procedures were conducted in accordance with the Guidelines on the Care and Use of Laboratory Animals issued by Shenyang Medical College. The protocol was approved by the ethical committee of Shenyang Medical College (Approval No. SYYXY2021042801).

### Statistical analysis

GraphPad Prism (GraphPad Software) was used for statistical analysis. Student's *t*-test was used to compare the differences between two groups. *P*-values <0.05 were considered statistically significant. For all the graphs in this study, error bars indicate the standard deviation of the duplicated or triplicated samples.

### Database

Gene expression profiles of CHB patients (GSE83148) (29–31) and HBV-infected human-liver chimeric mice (GSE52752), were downloaded from the Gene Expression Omnibus (GEO) public database. The expression level of EPN3 was extracted by GEO2R.

## Results

### EPN3 negatively regulates HBV RNA

**Figure 1:**
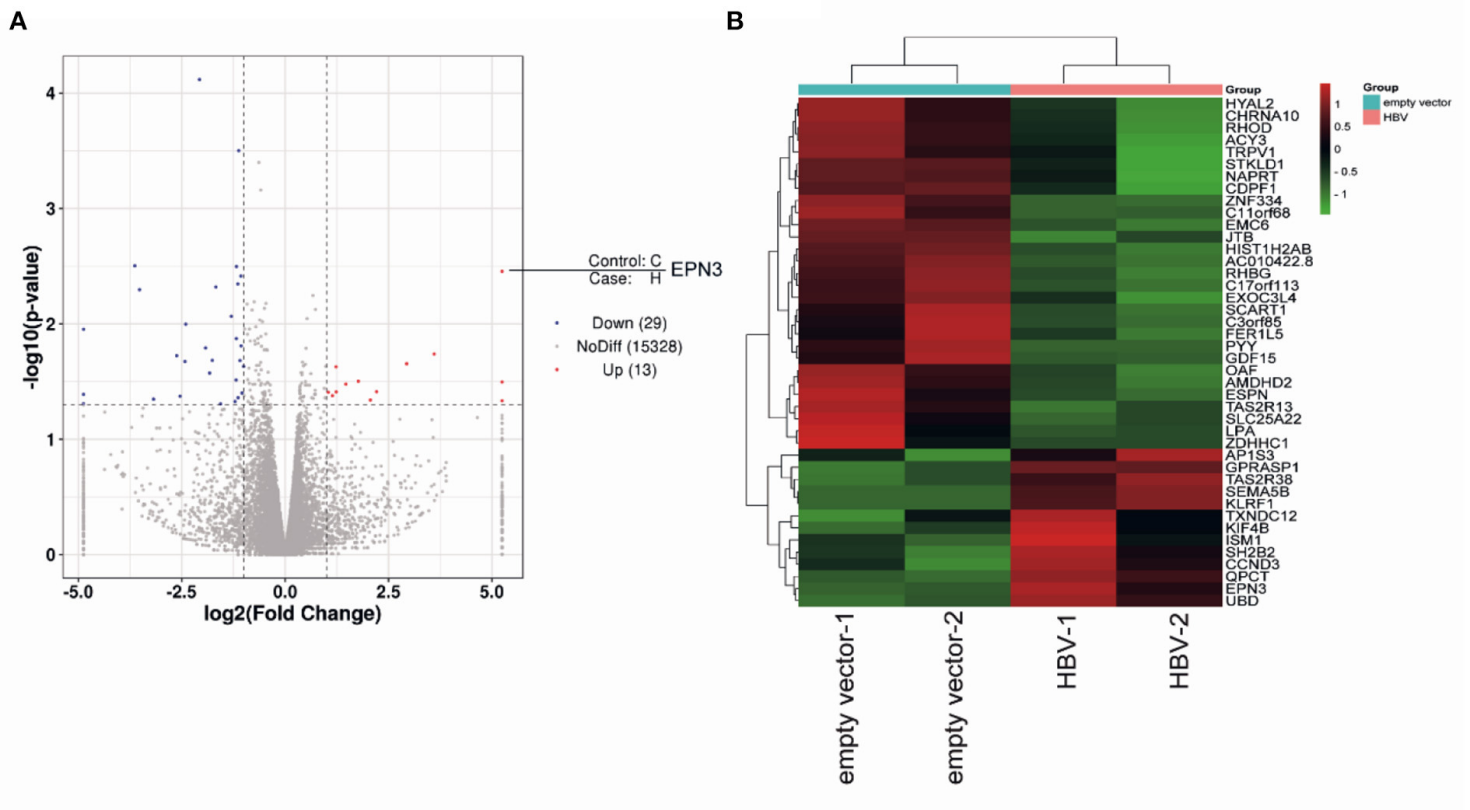
RNA-seq of pPB-transfected Huh7 cells. Huh7 cells were transfected with an HBV-replicon plasmid (pPB), or the empty vector. Three days after transfection, cells were harvested for RNA sequencing. The differentially expressed genes are indicated as a volcano plot **(A)** and a heatmap **(B)**.

**Figure 2:**
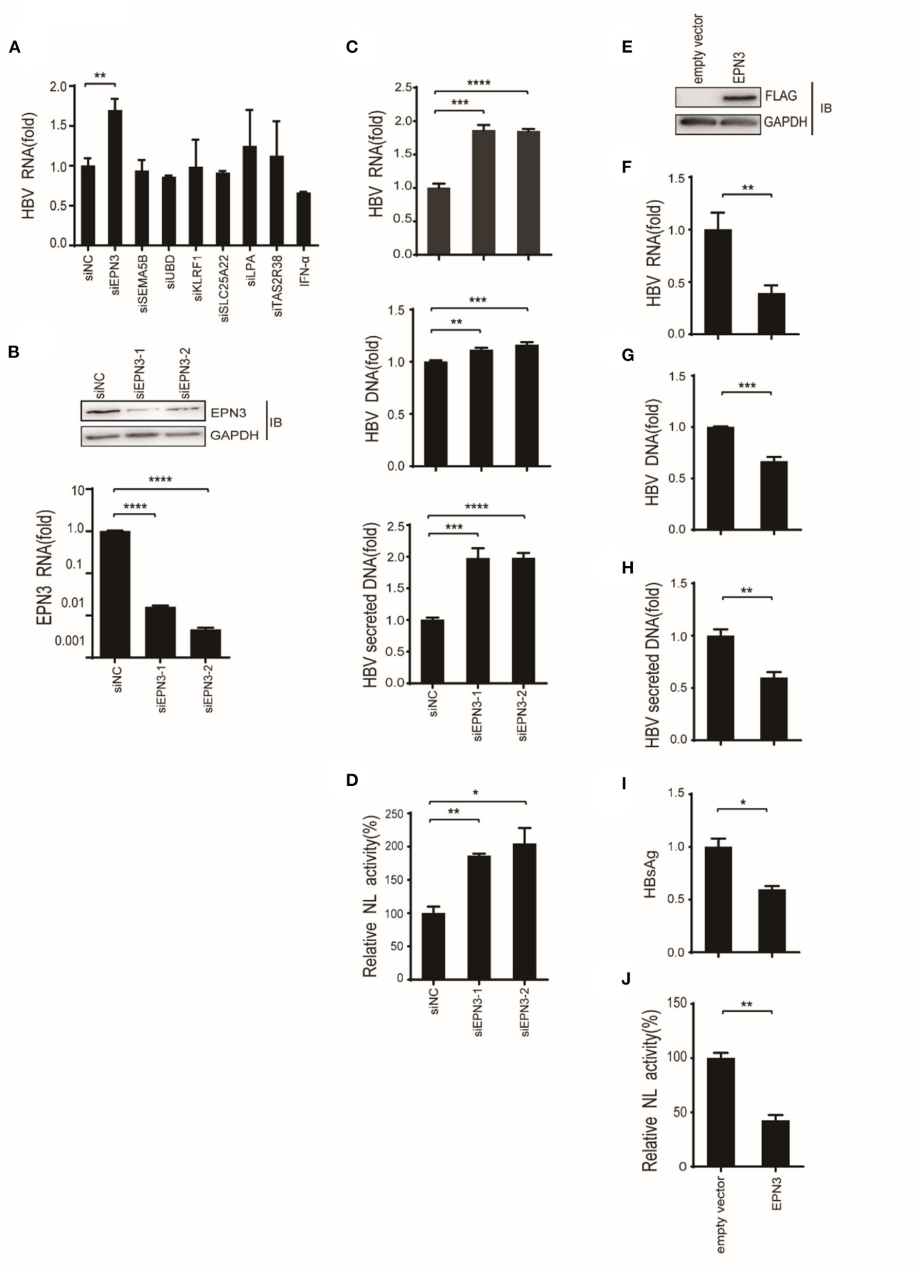
EPN3 negatively regulates the expression of HBV RNA. **(A–C)** Huh7 cells were transfected with pPB and the indicated siRNAs or the negative control (siNC), and 3 days later, the cells and supernatants were harvested. (**A,B** bottom, **C** top) HBV RNA and EPN3 mRNA were quantified by RT-qPCR analysis. (**B**, top) The total cellular lysates were subjected to western blot analysis. (**C**, middle and bottom) The cellular (middle) and secreted (bottom) HBV DNA levels were quantified by qPCR analysis. The level of siNC in the control sample is defined as value 1. **(D)** Cells were transfected with the pgRNA reporter (pCMV1.2xHBV/NL) and the helper plasmid (pcDNA-CP), and siEPN3. Three days later, cells were harvested and subjected to luciferase assay. The NL activity of siNC in the control sample is defined as value 100. (E-I) Cells were transfected with pPB and the FLAG-EPN3 expression vector, and harvested 72 h after transfection. **(E)** EPN3 overexpression was validated by Western blot. **(F–H)** The levels of cellular HBV RNA **(F)**, cellular HBV DNA **(G)**, and supernatant HBV DNA **(H)** were determined by RT-qPCR and DNA-qPCR. **(I)** The levels of supernatant HBsAg were measured by ELISA. The level of the empty vector is defined as value 1. **(J)** Cells were transfected with the EPN3, pgRNA reporter, and helper plasmids, and subjected to luciferase activity. The NL activity of the empty vector transfectant is defined as value 100. *P < 0.05, **P < 0.01, ***P < 0.001, ****P < 0.0001. Data are representative of two to three independent experiments.

To screen differential genes induced by HBV replication, Huh7 cells were transfected with an HBV replicon plasmid, pPB (15), followed by RNA-seq (Figures 1A,B). The analysis identified 148 host genes significantly expressed (*p* < 0.05), 13 and 29 of which were up-regulated or down-regulated more than 2-fold, respectively. To further verify their functional importance on viral replication, Huh7 cells were transfected with siRNAs targeting top 5 up-regulated (EPN3, SEMA5B, UBD, KLRF1, TAS2R38) and top 2 down-regulated genes (SLC25A22, LPA), along with pPB. The result revealed that the HBV-RNA level was significantly higher in the siEPN3 transfectant, compared to the siRNA Negative control (siNC) transfectant (Figure 2A). Other siRNAs did not affect viral RNA. The results were further validated by two individual siRNAs (Figure 2B), and the cellular and supernatant DNA were consistently upregulated (Figure 2C). In addition, making use of the HBV pgRNA reporter system (pCMV1.2xHBV/NL) (22, 23) where the nano-luciferase (NL) reflects the pgRNA level, we found that siEPN3 consistently increased the NL activity compared to the siNC (Figure 2D).

Further, Huh7 cells were co-transfected with a FLAG-tagged EPN3 expression vector and pPB (Figure 2E). We found that the cellular HBV RNA, the cellular and the supernatant viral DNA, and the supernatant HBsAg were decreased in the EPN3, but not EPN1 or EPN2 transfectant, compared to the empty vector (Figures 2F–I and Supplementary Figures 1, 2). The consistent result was obtained when we utilized another hepatic cell line, HepG2 cells (Supplementary Figure 3). In addition, EPN3 consistently reduced luciferase activity in the pCMV1.2xHBV/NL transfectant (Figure 2J).

**Figure 3:**
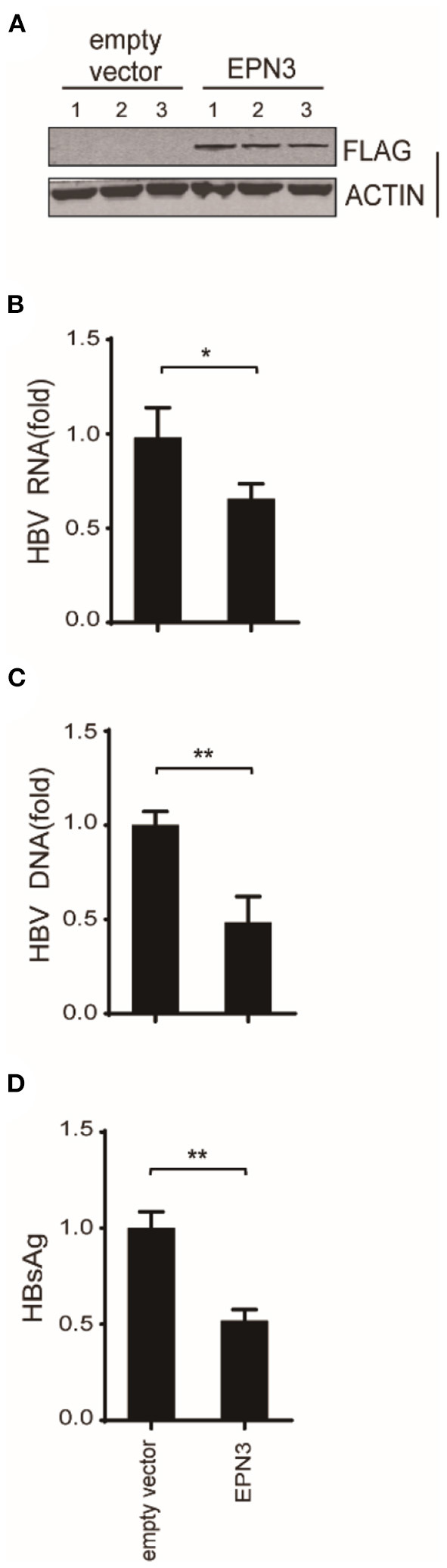
EPN3-mediated HBV RNA reduction in hydrodynamic-based mouse HBV model. C57BL/6 mice were hydrodynamically administrated with pPB and the FLAG-EPN3 or the empty vector (*n* = 3, each for the empty vector and EPN3). **(A)** Western blot analysis of the transfected liver. **(B)** HBV RNA levels were determined by RT-qPCR. **(C)** qPCR analyses of cytoplasmic HBV DNA. **(D)** The levels of HBsAg were measured in serum by ELISA. The level of the empty vector sample is defined as value 1. *P < 0.05, **P < 0.01.

And *in vivo*, by mining the microarray data of chronic hepatitis B (CHB) liver (GSE83148) (29–31) and HBV-infected human-liver chimeric mice (GSE52752), we found that EPN3 was highly expressed in the HBV-infected liver, compared to the uninfected controls (Supplementary Figure 4). And when HBV was overexpressed in murine liver making use of hydrodynamic injection (32), we found that hepatic viral RNA, DNA, and serum HBsAg were reduced compared to those injected with the empty vector (Figure 3). Overall, these results suggest that EPN3 negatively regulates HBV-RNA.

### EPN3 reduces HBV RNA in a transcription-coupled manner

**Figure 4:**
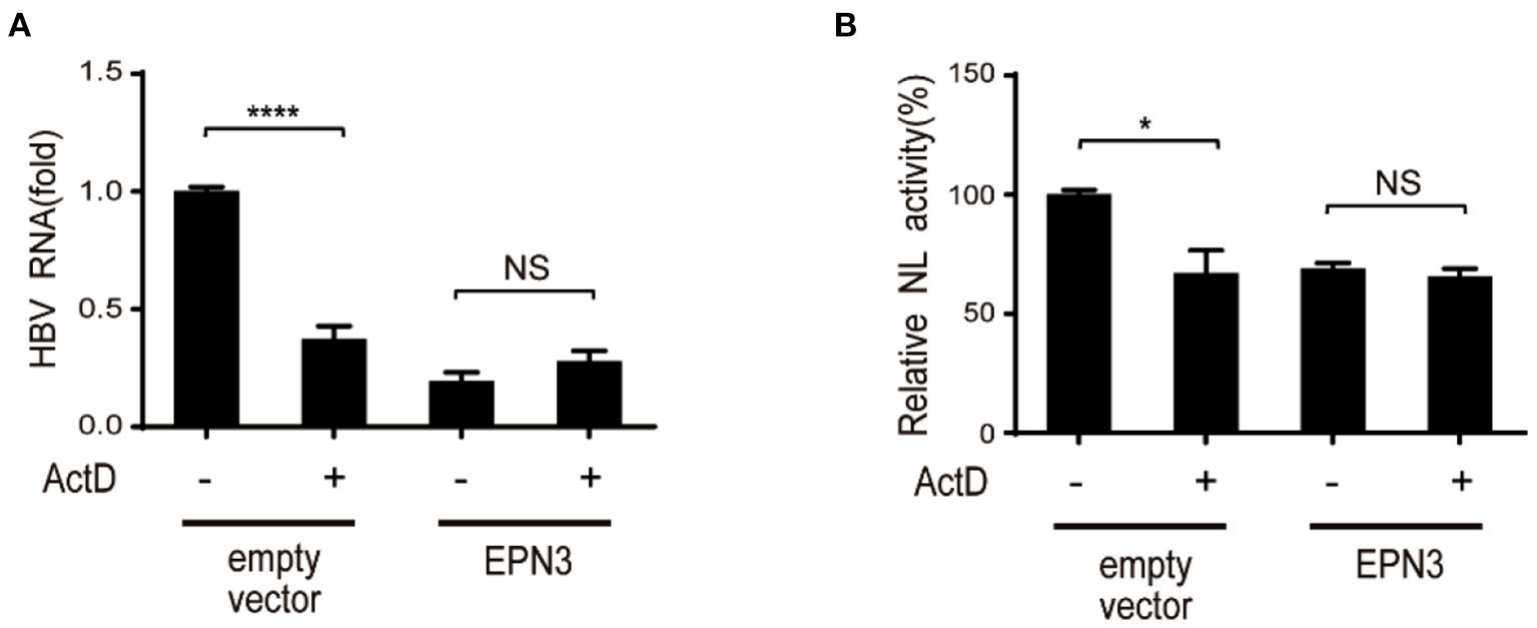
EPN3-mediated HBV downregulation depends on transcription. **(A)** Huh7 cells were transfected with pPB and the EPN3 expression vector. 54 h later, 100 ng/ml actinomycin D (ActD) was added for 18 h to block transcription. Total RNA was extracted to measure by RT-qPCR. The level of the empty vector without ActD treatment sample is defined as value 1. **(B)** Huh7 cells were transfected with EPN3, pCMV1.2xHBV/NL and pcDNA-CP. ActD was added 18 h before harvest, followed by luciferase assay. The NL activity of the empty vector without ActD treatment sample is defined as value 100. *P < 0.05, ****P < 0.0001. Data are representative of two to three independent experiments.

We further attempted to clarify the molecular mechanism by which EPN3 negatively regulates the expression of HBV RNA. We treated Huh7 cells co-transfected with pPB and the EPN3 expression vector, with a transcriptional inhibitor, actinomycin D (ActD). We found that ActD decreased viral RNA in the empty vector transfected, but not the pEPN3 transfectant (Figure 4). The result suggested that EPN3 reduced the viral RNA, dependent on its transcription, as we previously demonstrated for a host cytidine deaminase, Activation-Induced cytidine Deaminase (AID) (22, 26).

**Figure 5:**
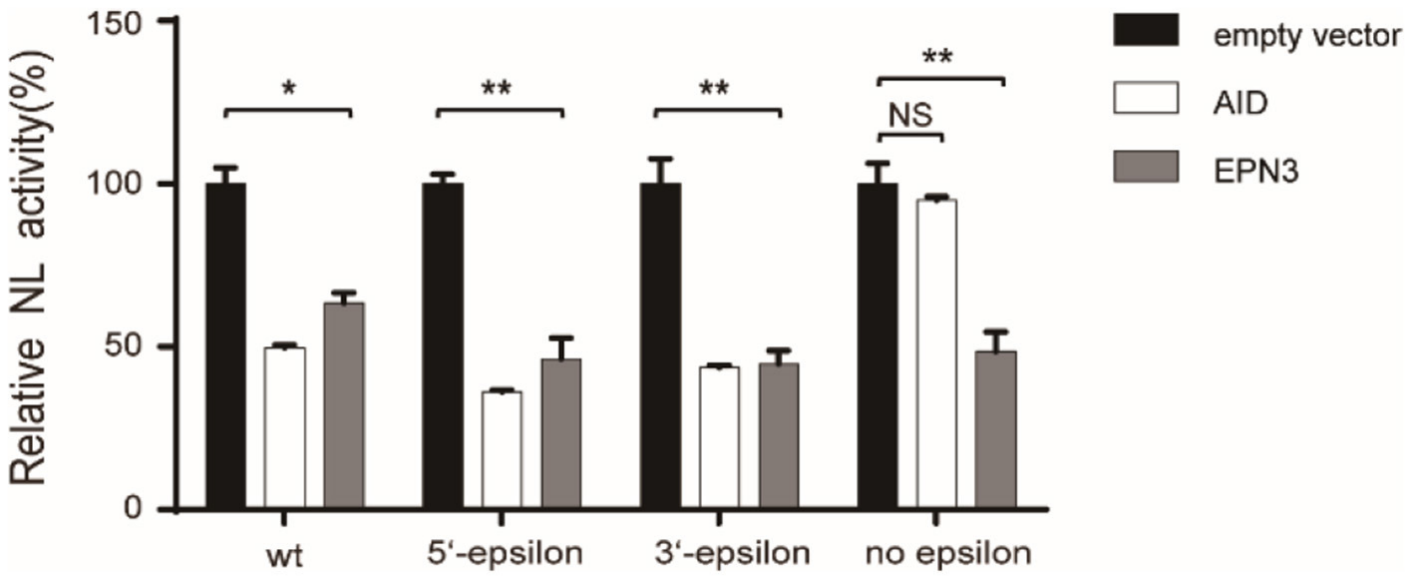
EPN3 does not require epsilon structures for its anti-HBV effect. Huh7 cells were transfected with the wildtype or mutated pCMV1.2xHBV/NL (WT: wild type, 5′-epsilon, 3′-epsilon, or no epsilon), along with pcDNA-CP and either empty, AID, or EPN3 expression vector. Three days after transfection, cells were harvested and luciferase activity was determined. The NL activity of the empty vector transfectants defined as value 100. *P < 0.05, **P < 0.01. Data are representative of two to three independent experiments.

Further, we verified the necessity of epsilon stem-loop structure for EPN3-mediated reduction of HBV RNA, which was important and essential for HBV replication and AID-mediated reduction of HBV RNA (22). We utilized mutant pCMV1.2xHBV/NL vectors, whose 5′- and/or 3′-epsilon structures were lacking. When the Huh7 cells were co-transfected with the mutant pCMV1.2xHBV/NL vectors and an AID expression vector, the NL activity was reduced in the transfectants lacking either 5′- or 3′-epsilon, but not both (Figure 5). In sharp contrast, EPN3 suppressed even the one deficient for both 5′- and 3′-epsilon, suggesting its distinct antiviral mechanisms from AID's.

### EPN3 suppresses HBV replication, downstream of p53 and IFN-α

**Figure 6:**
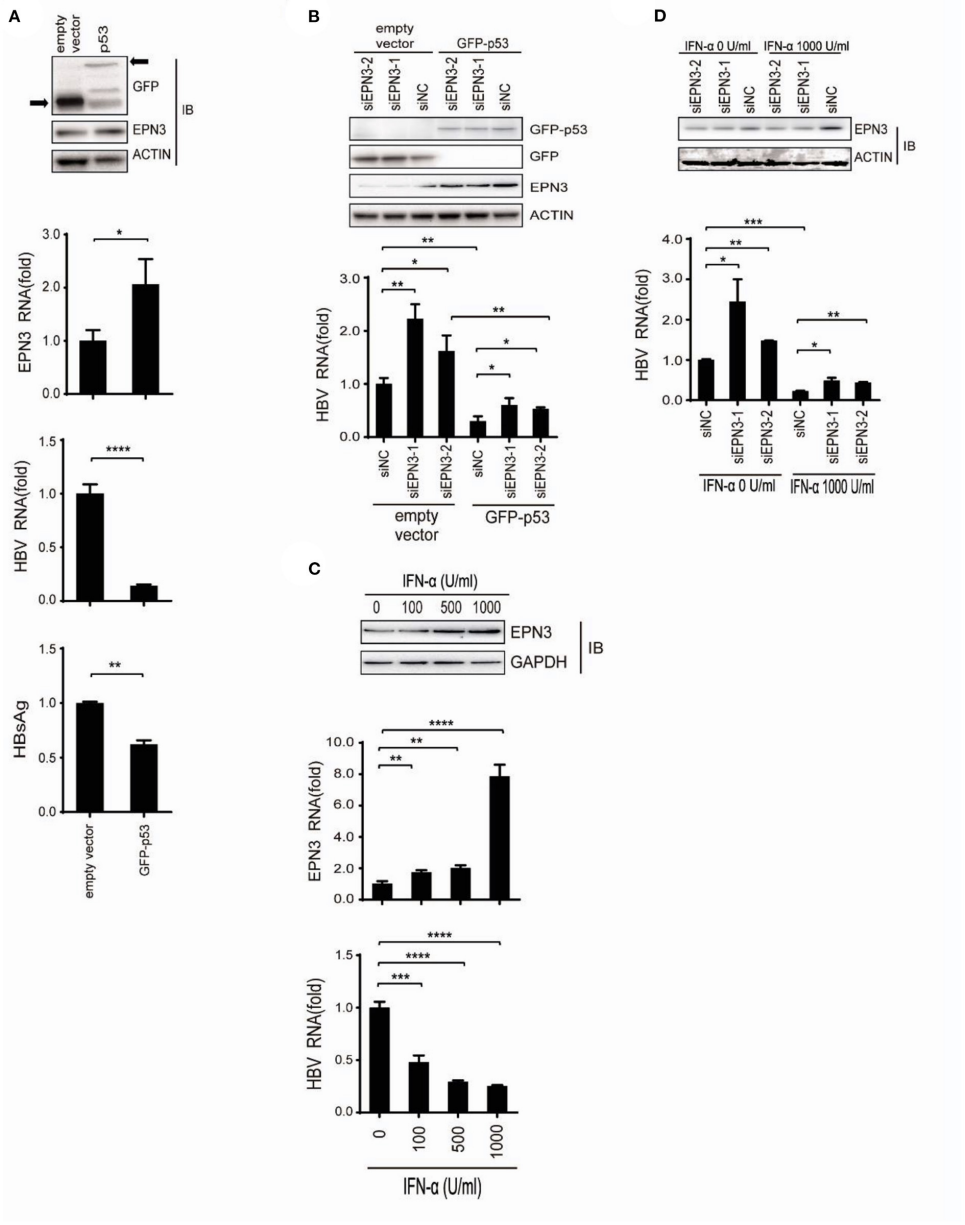
EPN3 suppresses HBV replication downstream of IFN-α and p53. **(A)** Huh7 cells were transfected with pPB and the p53 expression vector. Three days after transfection, cells were harvested and subjected to western blot (top) and RT-qPCR analysis (middle), and the culture supernatant was to ELISA (bottom). The level of the empty vector transfectant is defined as value 1. **(B)** Cells were transfected with pPB, the p53 expression vector, and siEPN3. Three days after transfection, cells were harvested for western blot (top) and RT-qPCR analysis (bottom). The level of siNC in the empty vector sample is defined as value 1. **(C)** The cells were transfected with pPB, and 6 h later, treated with IFN-α for 3 days. The expression level of EPN3 was determined using Western blot (top) and RT-qPCR analysis (middle). HBV RNA levels were determined by RT-qPCR (bottom). The level of untreated cells is defined as value 1. **(D)** Cells were transfected with pPB and siEPN3, and 6 h later, treated with 1,000 U/ml IFN-α for additional 3 days. The amount of EPN3 was determined using Western blot (top). HBV RNA levels were determined by RT-qPCR (bottom). The HBV level of the siNC transfectant, without IFN-α treatment is defined as value 1. *P < 0.05, **P < 0.01, ***P < 0.001, ****P < 0.0001. Data are representative of two to three independent experiments.

We further attempted to identify the pathophysiological context where EPN3 exerts its anti-HBV effect. We demonstrated that p53 reduced HBV (33), and Mori et al. reported that p53 up-regulated EPN3 in HCT116 cells (11). This led us to hypothesize that EPN3 is a downstream effector of p53-mediated anti-HBV pathway. Indeed, when we overexpressed HBV and GFP-p53, we found increased EPN3 expression and reciprocal HBV reduction, compared to the GFP transfectant (Figure 6A). In addition, when GFP-p53 and siEPN3 were co-transfected, the reduction of HBV RNA by GFP-p53 was attenuated by siEPN3 (Figure 6B), suggesting that EPN3 mediates anti-HBV activity of p53. IFN-α is one of the first-line anti-HBV drugs against chronic hepatitis B, inhibiting HBV replication (9, 33). We further investigated the role of EPN3 in the IFN-α-treated cells. When the Huh7 cells were treated with IFN-α, the expression of p53 and EPN3 was induced (Figure 6C top and middle and Supplementary Figure 5), while the HBV RNA was reciprocally reduced (Figure 6C bottom). Moreover, when the HBV replicon plasmid and the siEPN3 were co-transfected with Huh7 cells in the presence of IFN-α (Figure 6D), siEPN3 attenuated the reduction of viral RNA by IFN-α. Collectively, these results suggest that EPN3 negatively regulates the expression of HBV RNA, downstream of IFN-α/p53 pathway.

## Discussion

In this study, we investigated the role of a vesicle protein EPN3, in HBV infection. EPN3 RNA is upregulated by HBV (Figure 1 and Supplementary Figure 4), and knockdown of EPN3 increased HBV RNA in pPB-transfected Huh7 cells (Figures 2A–D), and its overexpression decreased, in the hepatic cell lines and mice (Figures 2E–J, 3 and Supplementary Figures 1–3). The EPN3-mediated viral reduction was dependent on transcription, but not the epsilon structure of viral RNA (Figures 4, 5). And the EPN3-mediated anti-HBV effect was exerted downstream of IFN-α and p53 (Figure 6). This study demonstrates the role of EPN3, as an antiviral effector of IFN-α and p53.

We previously reported that AID recruits RNA degradation complex (RNA Exosome) to the epsilon structure of HBV RNA, which is degraded in a transcription-coupled manner (26). In addition, Mao et al. reported that zinc finger antiviral protein (ZAP), which promotes viral RNA decay, targets the terminal redundant region and epsilon structure (22, 34). In sharp contrast, EPN3 did not require the epsilon structure for its antiviral activity (Figure 5), suggesting a distinct mechanism from AID and ZAP. The exact molecular mechanism by which EPN3 negatively regulates viral RNA remains to be determined, including whether it affects viral promoter activity or viral RNA stability.

Epsins are ubiquitin-binding endocytic proteins, and modulates many signaling pathways including Notch, Rho, and VEGFR (35). Their expression is associated with low relapse-free survival in ER-negative breast cancer (36), and EPN3 reportedly induced cell migration and invasion (37). In association with p53, Mori et al. reported that EPN3 is a key mediator of apoptosis downstream of p53 (11). Compared to their roles in tumorigenesis, those in viral infection have not been investigated. Indeed, this study demonstrates the antiviral role of Epsins, downstream of IFN-α and p53 (Figure 6) and its exact mechanism remains to be determined. Of note, EPN3 reportedly increased E-cadherin endocytosis, and induced EMT expressed in the clathrin-coated vesicles and shuttled to the nucleus in keratinocytes (38). Given that the finding can be extended to hepatocytes, it is intriguing to speculate that EPN3 regulates the amount of host factors in the nucleus, important for the HBV core promoter activity and/or viral RNA stabilization.

This study has several limitations; Firstly, the exact molecular mechanism how EPN3 negatively regulates the expression of HBV RNA remains to be determined, especially whether it affects core promoter activity or RNA stability; Secondly, upregulation of EPN3 protein in vivo, by HBV infection remains to be verified. In addition, contribution of IFN-α-p53-EPN3 pathway to HBV elimination should be determined utilizing animal models, such as human liver chimeric mice.

In conclusion, we identified a novel host factor, EPN3, negatively regulating the expression of HBV RNA downstream of IFN-α-p53 pathway. Further studies are warranted to clarify the detailed molecular mechanism, and distinguish the anti-viral effect of IFN-α from unwanted side effects, and pave the way for more effective therapies with little side effects.

## Data availability statement

 The data presented in the study are deposited in the BioProject database of the National Center for Biotechnology Information (Accession number PRJNA856828).

## Ethics statement

The animal study was reviewed and approved by the Ethical Committee of Shenyang Medical College (Approval No. SYYXY2021042801).

## Author contributions

GL: study conception and design. XL, CD, XX, FC, YinZ, and YupZ: data acquisition. XL, WZ, XF, YunZ, YS, BY, YB, BW, and YL: data analysis and interpretation. GL, XL, MM, and KW: manuscript preparation. GL: study supervision. All authors contributed to the article and approved the submitted version.

## Funding

This work was funded by grants from the National Natural Science Foundation of China (81702856).

## Conflict of interest

The authors declare that the research was conducted in the absence of any commercial or financial relationships that could be construed as a potential conflict of interest.

## Publisher's note

All claims expressed in this article are solely those of the authors and do not necessarily represent those of their affiliated organizations, or those of the publisher, the editors and the reviewers. Any product that may be evaluated in this article, or claim that may be made by its manufacturer, is not guaranteed or endorsed by the publisher.
