# Supplementary material for: Interferon-alpha responsible EPN3 regulates hepatitis B virus replication
Source: Front Med (Lausanne). 2022 Jul 22;9:944489. doi: 10.3389/fmed.2022.944489 (PMC9354525; doi:10.3389/fmed.2022.944489)
Supplement: Supplementary file 1 [file Table_1.DOCX]

**Supplementary Table 1: The information of plasmid**

| Plasmid name | Origin |
| --- | --- |
| pCMV-AID-GFP | (Liang et al. 2015) |
| pcDNA-CP | (Que et al. 2017) |
| pCMV1.2xHBV/NL wt | (Que et al. 2017) |
| pCMV1.2xHBV/NL-5’ε | (Que et al. 2017) |
| pCMV1.2xHBV/NL-no ε | (Que et al. 2017) |
| pCMV1.2xHBV/NL-3’ε | (Que et al. 2017) |
| p3xFLAG-EPN3-CMV-10 | The cDNA of EPN3 was subcloned into p3xFLAG-CMV-10 expression vector. |
| pPB | (Kim et al. 2004, Liang et al. 2015) |
| EGFP-p53 | (Liu et al. 2019) |
| p3xFLAG-EPN1-CMV-10 | The cDNA of EPN1 was subcloned into p3xFLAG-CMV-10 expression vector. |
| p3xFLAG-EPN2-CMV-10 | The cDNA of EPN2 was subcloned into p3xFLAG-CMV-10 expression vector. |
