# Supplementary material for: Interferon-alpha responsible EPN3 regulates hepatitis B virus replication
Source: Front Med (Lausanne). 2022 Jul 22;9:944489. doi: 10.3389/fmed.2022.944489 (PMC9354525; doi:10.3389/fmed.2022.944489)
Supplement: Supplementary file 2 [file Table_2.docx]

**Supplementary Table 2: The information of primer for qPCR.**

| Target gene |  | sequence | Target sequence | Accession No. | Length of  the target | Reference |
| --- | --- | --- | --- | --- | --- | --- |
| EPN3 | F | GAGAAGCTAAAAACCAGCCAGT | **gagaagctaaaaaccagccagt**cctccatcctggacttggctgacatcttcgtacctgccctggccccgccc**tccacacactgctctgctg** | NM_017957.3 | 91bp | (Mori et al. 2017) |
|  | R | CAGCAGAGCAGTGTGTGGA |  |  |  |  |
| HBV | F | GAATTGATGACTCTAGCTACCTG | **gagttgatgaatctagccacctg**ggtgggaagtaatttggaagacccagcctcccgggaattagtagtcagttatgtcaatgttaatatgggcctaaa**aatcagacaactattgtggtttc** | M54923.1 | 121bp | (Liang et al. 2015, Liu et al. 2019, Li et al. 2020) |
|  | R | GAAACCACAATAGTTGCCTGATC |  |  |  |  |
| β-actin | F | CTGAGGCACTCTTCCAGCCTTCCTT | **ctgaggcactcttccagcctt**ccttcctgggcatggagtcctgtggcatccacgaaactaccttcaactccatcatgaagtgtgacgtggacatccgcaaagacctgtacgccaacacagtgctgtctggcggcaccaccatgtaccctggcattgccgacag**gatgcagaaggagatcactgccctg** | NM_001101.5 | 188bp | This tudy. |
|  | R | CAGGGCAGTGATCTCCTTCTGCATG |  |  |  |  |
| m-β-actin | F | CTGGAGAAGAGCTATGAGCTGC | **ctggagaagagctatgagctgc**ctgacggccaggtcatcactattggcaacgagcggttccgatgccctgaggctcttttccagccttccttcttgggtatggaatcctgtggcatccatgaaactacattcaatt**ccatcatgaagtgtgacgttg** | NM_007393.5 | 157bp | (Liang et al. 2015) |
|  | R | CAACGTCACACTTCATGATGG |  |  |  |  |
| p53 | F | AGGCCTTGGAACTCAAGGAT | **aggccttggaactcaaggat**gcccaggctgggaaggagccaggggggagcagggctcactccagc**cacctgaagtccaaaaaggg** | NM_000546.6 | 85bp | (Liu et al. 2019) |
|  | R | CCCTTTTTGGACTTCAGGTG |  |  |  |  |
