# Supplementary material for: Interferon-alpha responsible EPN3 regulates hepatitis B virus replication
Source: Front Med (Lausanne). 2022 Jul 22;9:944489. doi: 10.3389/fmed.2022.944489 (PMC9354525; doi:10.3389/fmed.2022.944489)
Supplement: Supplementary file 3 [file Data_Sheet_1.docx]

**Supplementary Fig.1. Detect EPN3 inhibiting HBV replication in different time.**

Huh7 cells were transfected with pPB and the EPN3 expression vector. Cells were harvested at the indicated time points. HBV RNA levels were determined by RT-qPCR. Values of the empty vector transfectant are defined as value 1. *, *P*<0.05, **, *P*<0.01. Data are representative of three independent experiments.

**Supplementary Fig.2. EPN3 but not EPN1 and EPN2 blocked HBV replication.**

Huh7 cells were transfected with pPB and the EPN1, EPN2, or EPN3 expression vector, and were harvested 72 hours later. HBV RNA levels were determined by RT-qPCR. Values of the empty vector transfectant are defined as value 1. ***, *P*<0.001. Data are representative of three independent experiments.

**Supplementary Fig.3. EPN3 blocks HBV replication in HepG2 cells.**

HepG2 cells were transfected with pPB and the EPN3 expression vector. Cells were harvested at 72 hours. (A) EPN3 overexpression was validated by Western blot. (B) HBV RNA levels were determined by RT-qPCR. (C, D) HBV DNA levels in the cytoplasm or culture supernatant were determined by qPCR analysis. Values of the empty vector transfectant are defined as value 1. *, *P*<0.05. Data are representative of three independent experiments.

**Supplementary Fig.4.** **EPN3 mRNA level in CHB and human liver-chimeric mice.**

(A, B) mRNA levels of EPN3 in liver tissues from patients with chronic hepatitis B (CHB) (A, GSE83148, CHB: n=122, healthy controls: n=6), and human liver-chimeric mice (B, GSE52752, HBV-infected: n=16, control: n=6).

**Supplementary Fig.5. IFN-α increases p53 expression level in Huh7 cells.**

Huh7 cells were transfected with pPB, and six hours after transfection, 1000 U/ml IFN-α was added. Three days later, the cells were subjected to RT-qPCR. The value of IFN-α (-) cells is defined as value 1. ***, *P*<0.001. Data are representative of three independent experiments.
