# Supplementary figures and images for: Interferon-alpha responsible EPN3 regulates hepatitis B virus replication
Source: Front Med (Lausanne). 2022 Jul 22;9:944489. doi: 10.3389/fmed.2022.944489 (PMC9354525; doi:10.3389/fmed.2022.944489)

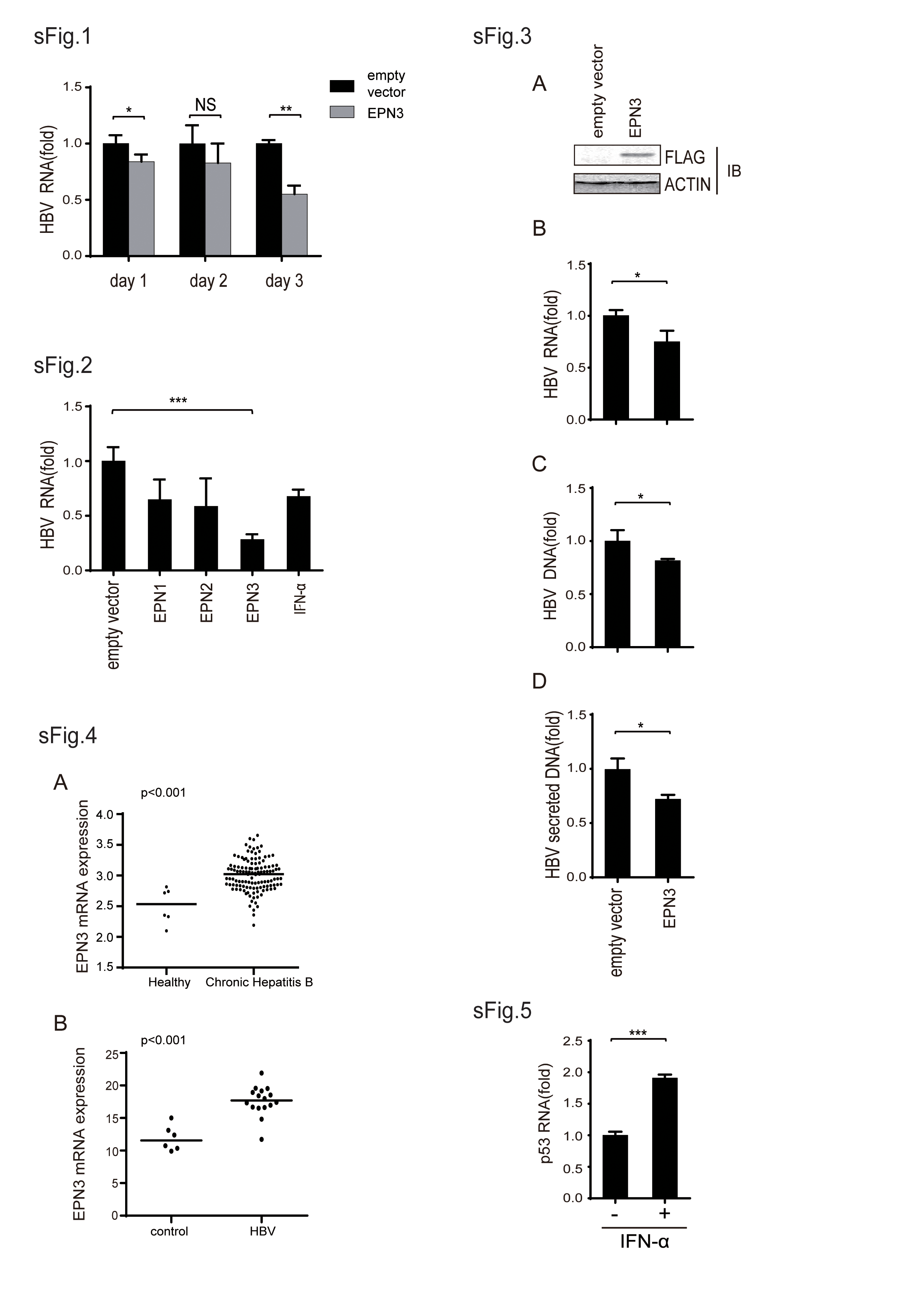

Supplement: Supplementary file 4 [file Image_1.TIF]
